# Stimulatory Effects of Melatonin on Porcine In Vitro Maturation Are Mediated by MT2 Receptor

**DOI:** 10.3390/ijms19061581

**Published:** 2018-05-26

**Authors:** Sanghoon Lee, Jun-Xue Jin, Anukul Taweechaipaisankul, Geon-A Kim, Byeong-Chun Lee

**Affiliations:** Department of Theriogenology and Biotechnology, College of Veterinary Medicine, Seoul National University, 1 Gwanak-ro, Gwanak-gu, Seoul 08826, Korea; sodany2@snu.ac.kr (S.L.); junxue-jin@hotmail.com (J.-X.J.); famfamat@gmail.com (A.T.); pshsje03@snu.ac.kr (G.-A.K.)

**Keywords:** melatonin membrane receptor, MT2, melatonin, porcine, in vitro maturation, cumulus expansion

## Abstract

Melatonin is a multifunctional molecule with numerous biological activities. The fact that melatonin modulates the functions of porcine granulosa cells via the MT2 receptor suggests the possibility of MT2 receptor-mediation for melatonin to promote cumulus expansion of porcine cumulus-oocyte complexes (COCs). Therefore, we investigated the presence of MT2 in porcine COCs, and the effects of melatonin with or without selective MT2 antagonists (luzindole and 4-P-PDOT) on this process; COCs underwent in vitro maturation culturing with six different conditions (control, melatonin, luzindole, 4-P-PDOT, melatonin + luzindole or melatonin + 4-P-PDOT). Cumulus expansion, oocyte nuclear maturation, and subsequent embryo development after parthenogenetic activation (PA) were evaluated. In experiment 1, MT2 was expressed in both oocytes and cumulus cells. In experiment 2, melatonin significantly increased the proportion of complete cumulus expansion (degree 4), which was inhibited by simultaneous addition of either luzindole or 4-P-PDOT. A similar pattern was observed in the expression of genes related to cumulus expansion, apoptosis, and *MT2*. In experiment 3, no significant difference was observed in immature, degenerate, and MII oocyte rates among the groups. In experiment 4, melatonin significantly increased blastocyst formation rates and total blastocyst cell numbers after PA, but these effects were abolished when either luzindole or 4-P-PDOT was added concomitantly. In conclusion, our results indicate that the MT2 receptor mediated the stimulatory effects of melatonin on porcine cumulus expansion and subsequent embryo development.

## 1. Introduction

Melatonin (*N*-acetyl-5-methoxytryptamine), a natural neurohormone synthesized by the mammalian pineal gland, is involved in the regulation of the circadian rhythm [1]. In addition, many other tissues and cells, including bone marrow [2], lymphocytes [3], retina [4], astrocytes [5], thymus [6], and female reproductive organs (granulosa cells, cumulus cells and oocytes) [7], can synthesize melatonin. Evidence indicates that melatonin exists in follicular fluid [8], and that it could be synthesized by the oocytes, as well as that taken up from the blood circulation [9,10]. In women, melatonin was suggested as an efficient predictor of positive in vitro fertilization outcomes, and as a medication to enhance oocyte and embryo quality in patients with infertility [11,12].

Melatonin is a potent free radical scavenger and antioxidant [13]; the free radical scavenging activity of melatonin and its metabolites efficiently protects against oxidative stress [14,15]. In addition to direct free radical scavenging, some actions of melatonin are mediated by its two high-affinity G protein-coupled receptors, MT1 and MT2 [16,17]. Although multiple actions of melatonin on a number of different physiological processes could be mediated by its antioxidant activity in scavenging free radicals or by melatonin membrane receptors, previous studies explained the stimulatory effects of melatonin on in vitro maturation (IVM) of oocytes mainly by its antioxidant effects [8,18,19]. However, a direct action of melatonin on porcine cumulus oocyte complexes (COCs) through melatonin membrane receptors remains unproven. Recently, the existence of melatonin membrane receptors has been shown in the ovaries of mammals [11].

The follicle is the structural and functional unit of the mammalian ovary; it is composed of antral and mural granulosa cell layers attached to the follicular wall, enclosing an oocyte surrounded by cumulus cells [20]. Cumulus cells and granulosa cells were demonstrated to play important roles in metabolic support and follicular steroidogenesis [21,22]. Thus, they are involved in establishing an essential microenvironment for the follicle-enclosed oocyte in coordination with endocrine, paracrine, and autocrine signals [23].

Recently, we reported that melatonin increases porcine cumulus expansion in vitro and subsequent embryo development through the activation of sonic hedgehog signaling [24]. In addition, the presence of mRNAs of melatonin membrane receptors MT1 and MT2 in cumulus cells of pigs was demonstrated [24]. The presence of melatonin membrane receptors in cumulus cells infers that the effects of melatonin on cumulus expansion might be mediated by these receptors. As expansion of the granulosa and cumulus cell layers—which is regulated by hedgehog signaling [25]—is an important process for oocyte maturation [26], the fact that melatonin modulates the functions of porcine granulosa cell through the MT2 receptor [27] suggests that this receptor is likely to mediate the effects of melatonin on porcine cumulus expansion. Therefore, we hypothesized that melatonin could exert stimulatory effects on porcine cumulus expansion through the MT2 receptor. To investigate this, we used selective MT2 antagonists that show substantially higher affinities for MT2 receptors, including luzindole (11 fold) and 4-phenyl-2-propionamidotetralin (4-P-PDOT) (61 fold) [28,29].

Although several studies revealed that melatonin stimulates IVM of oocytes in pigs, whether the underlying mechanism by which melatonin promotes porcine cumulus expansion is receptor-mediated has not been investigated. The aim of this study, therefore, was to investigate whether the effects of melatonin on porcine cumulus expansion are mediated by the MT2 receptor. In this study, we compared the effects of melatonin with or without selective MT2 antagonists (luzindole or 4-P-PDOT) on cumulus expansion, oocyte nuclear maturation, and subsequent embryonic development.

## 2. Results

### 2.1. Detection of Melatonin Membrane Receptor 2 (MT2) in Porcine Cumulus-Oocyte Complexes

**Figure 1:**
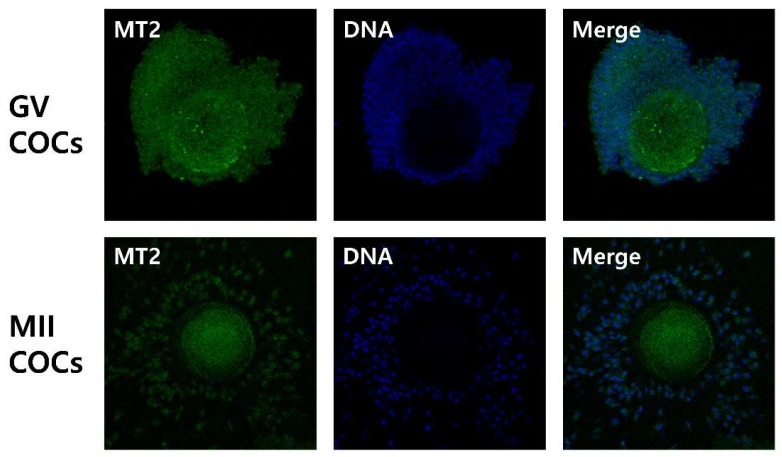
Immunofluorescence images of melatonin receptor 2 (MT2) in porcine germinal vesicle (GV) and Metaphase II (MII) cumulus-oocyte complexes (COCs). Porcine COCs were incubated with MT2 antibodies followed by FITC-conjugated rabbit anti-goat IgG (green). DNA was counterstained with Hoechst-3334. Original magnification 100×.

In the first experiment, the expression of the MT2 receptor protein in porcine COCs was investigated. Immunofluorescence analysis revealed the presence of the MT2 receptor protein in porcine germinal vesicle (GV) and metaphase II (MII) stage COCs (Figure 1).

### 2.2. Effects of Melatonin with or without Selective MT2 Antagonists on Cumulus Expansion

**Figure 2:**
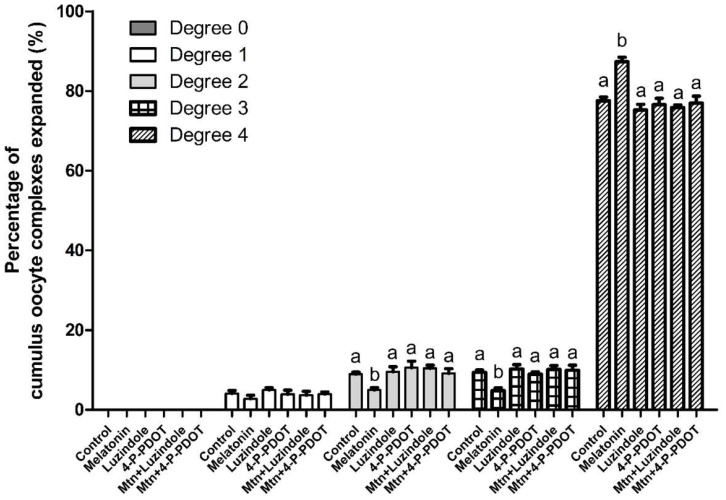
Effects of 10^−9^ M melatonin with or without selective MT2 antagonists (10^−9^ M luzindole or 10^−9^ M 4-P-PDOT) on cumulus expansion at 44 h of IVM. The degree of cumulus expansion was classified into five groups, as described previously [30]. A total of 1344 cumulus-oocyte complexes was used in six independent replicates. Data are shown as the means ± SEM. Within each category, groups marked with different letters are significantly different (*p* < 0.05). Mtn, melatonin.

**Figure 3:**
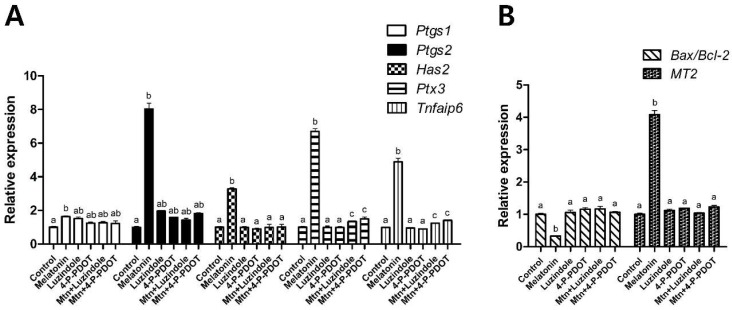
Effects of 10^−9^ M melatonin with or without selective MT2 antagonists (10^−9^ M luzindole or 10^−9^ M 4-P-PDOT) on gene expression in porcine cumulus cells. (**A**) Cumulus expansion genes (*Ptgs1*, *Ptgs2*, *Has2*, *Ptx3* and *Tnfaip6*); (**B**) Apoptosis gene (*Bax/Bcl-2* ratio) and *MT2*. Data are shown as the means ± SEM. Within each category, groups marked with different letters are significantly different (*p* < 0.05). The experiment was replicated at least three times. Mtn, melatonin.

In experiment 2, the effects of 10^−9^ M melatonin on cumulus expansion were investigated with or without the selective MT2 antagonists (10^−9^ M luzindole or 10^−9^ M 4-P-PDOT) treatment during IVM (Figure 2). Melatonin significantly increased the proportion of COCs exhibiting complete cumulus expansion (degree 4) (melatonin, 87.4% vs. control, 77.5%), and decreased the proportion of degrees 2 and 3 compared to the control (melatonin, 5.0% and 4.8% vs. control, 9.0% and 9.4%, respectively). Neither of the selective MT2 antagonists (lunzidole or 4-P-PDOT) treatments affected the degree of cumulus expansion. However, when either luzindole or 4-P-PDOT was added simultaneously with melatonin, they abolished the effect of melatonin on the degree of cumulus expansion. In terms of expression of genes associated with cumulus expansion, apoptosis, and *MT2*, a similar pattern was observed (Figure 3). Melatonin significantly increased the expression of cumulus expansion genes (*Ptgs1*, *Ptgs2*, *Has2*, *Ptx3* and *Tnfaip6*) and *MT2*, and decreased the expression of the apoptosis gene (*Bax*/*Bcl-2* ratio) compared to the control. However, when either luzindole or 4-P-PDOT was included simultaneously with melatonin, the genes whose expression was changed by melatonin were not affected.

### 2.3. Effects of Melatonin with or without Selective MT2 Antagonists on Oocyte Nuclear Maturation

**Figure 4:**
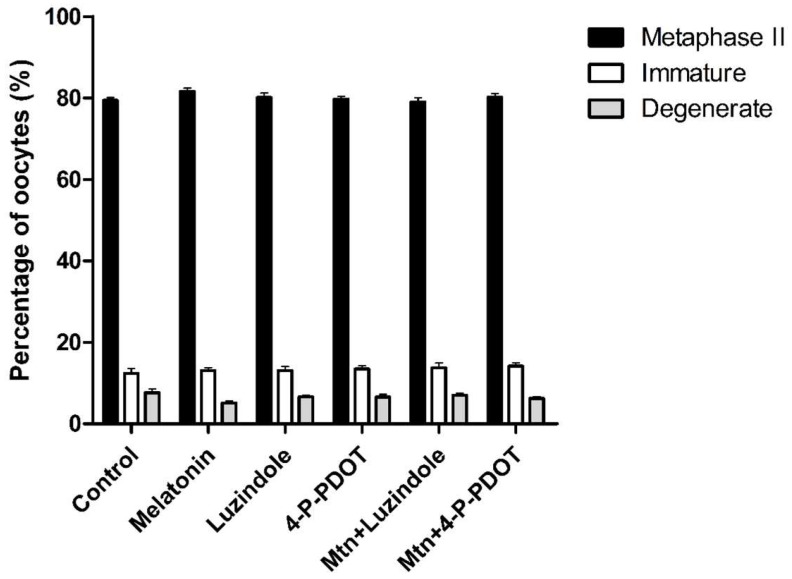
Effects of 10^−9^ M melatonin with or without selective MT2 antagonists (10^−9^ M luzindole or 10^−9^ M 4-P-PDOT) on the nuclear maturation of porcine oocytes. A total of 1632 oocytes was used in seven independent replicates. Data are shown as the means ± SEM. There was no effect of melatonin with or without selective MT2 antagonists on oocyte nuclear maturation. Mtn, melatonin.

In experiment 3, the effects of 10^−9^ M melatonin treatment with or without selective MT2 antagonists (10^−9^ M luzindole or 10^−9^ M 4-P-PDOT) during IVM on oocyte nuclear maturation were investigated (Figure 4). Melatonin treatment showed no differences in MII, immature, and degeneration rates, compared to the control. Also, neither luzindole nor 4-P-PDOT treatment affected oocyte nuclear maturation. Even when either luzindole or 4-P-PDOT was added concomitantly with melatonin, oocyte nuclear maturation was not affected.

### 2.4. Effects of Melatonin with or without Selective MT2 Antagonists Treatment during IVM on Subsequent Development of PA Embryos

**Figure 5:**
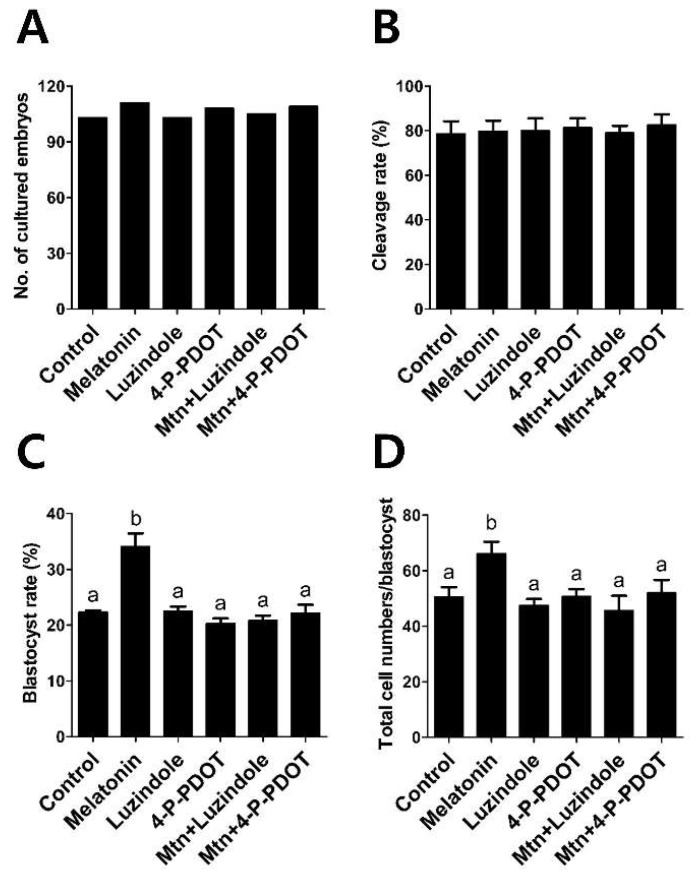
Effects of 10^−9^ M melatonin with or without selective MT2 antagonists (10^−9^ M luzindole or 10^−9^ M 4-P-PDOT) during IVM on subsequent embryo development after parthenogenetic activation. (**A**) Number of cultured embryos; (**B**) Cleavage rate; (**C**) Blastocyst rate; (**D**) Total cell numbers/blastocyst. A total of 639 oocytes was used in four independent replicates. Data are shown as the means ± SEM. Within each category, groups marked with different letters are significantly different (*p* < 0.05). Mtn, melatonin.

In experiment 4, we evaluated the effects of 10^−9^ M melatonin treatment with or without selective MT2 antagonists (10^−9^ M luzindole or 10^−9^ M 4-P-PDOT) during IVM on subsequent embryonic development after PA (Figure 5). The melatonin treatment group showed a significant increase in blastocyst formation rates and total cell numbers, compared with the control (melatonin, 34.1% and 66.2 vs. control, 22.3% and 50.5, respectively). Neither the luzindole nor the 4-P-PDOT group showed a difference in blastocyst formation rates and total cell numbers, compared to the control (luzindole, 22.4% and 47.5; 4-P-PDOT, 20.3% and 50.7, respectively). However, when either luzindole or 4-P-PDOT was included concomitantly with melatonin, they abolished the stimulatory effect of melatonin on blastocyst formation rates and total cell numbers (melatonin plus luzindole, 20.8% and 45.7; melatonin plus 4-P-PDOT, 22.1% and 52.0, respectively).

## 3. Discussion

Modulation of functions of porcine granulosa cells by melatonin via the MT2 receptor was demonstrated in a recent study [27]. However, whether the stimulatory effects of melatonin on porcine IVM are meditated by the MT2 receptor was not investigated. In this report, for the first time, we demonstrated that a melatonin-induced improvement in cumulus expansion, expression of genes related to cumulus expansion, apoptosis, and *MT2* in cumulus cells and subsequent embryo development was prevented by either of the two selective MT2 receptor antagonists (luzindole or 4-P-PDOT). Therefore, our data suggest that the ability of melatonin to promote cumulus expansion and subsequent embryo development is mediated by the MT2 receptor.

As in other organs, some effects of melatonin on the ovary are dependent on conventional membrane receptors (MT1/MT2) [31]. The ovarian follicle is comprised of somatic cells (cumulus cell and granulosa cell layers) surrounding an oocyte [20]. Expansion of the granulosa and cumulus cell layers plays an important role in oocyte maturation [26], and facilitates ovulation in the ovulatory follicle [20]. Recently, we demonstrated that the mRNA of melatonin receptors MT1 and MT2 are expressed in porcine cumulus cells [24]. The presence of melatonin membrane receptors in cumulus cells and the evidence of MT2 receptor-mediated modulation in porcine granulosa cell functions [27] infers that the MT2 receptor could mediate the effects of melatonin on cumulus expansion. To our knowledge, this is the first report to demonstrate the presence of the MT2 receptor protein in porcine GV and MII stage COCs (Figure 1).

The culture conditions during oocyte maturation can dramatically influence the degree of cumulus expansion, the meiotic maturation of oocytes, and the subsequent developmental competence of embryos derived from them [32]. Changes in cumulus expansion and nuclear maturation of porcine COCs are directly associated with subsequent developmental competence of oocytes [33]. Therefore, we evaluated cumulus expansion, nuclear maturation of oocytes, and subsequent embryo development, to investigate whether the MT2 receptor mediates the stimulatory effects of melatonin on IVM, using the selective MT2 antagonists (luzindole or 4-P-PDOT).

Cumulus expansion of porcine COCs was significantly increased by melatonin treatment. Cumulus expansion has been used as a morphological indicator to predict the quality of oocytes following maturation [34], because optimum expansion of the cumulus cell layers is required for proper oocyte maturation [35]. However, when either of the selective MT2 antagonists was added concomitantly with melatonin, the melatonin-induced increase in cumulus expansion was abolished. These results suggested that the ability of melatonin to promote cumulus expansion is likely mediated by the MT2 receptor. To further clarify the mechanisms by which melatonin exerts its stimulatory effects on cumulus expansion, we investigated the expression of genes related to cumulus expansion, apoptosis, and *MT2* in cumulus cells. Cumulus expansion genes (*Ptgs1*, *Ptgs2*, *Has2*, *Ptx3* and *Tnfaip6*) which regulate cumulus expansion during oocyte maturation, and *MT2*, the transcript of melatonin receptor 2, were up-regulated in the melatonin-treated group. An increased expression of cumulus expansion genes and *MT2* suggests that MT2 receptor might be responsible for melatonin-induced cumulus expansion. The apoptosis gene (*Bax/Bcl-2* ratio), which is an indicator of apoptosis [36,37], was decreased by melatonin. This indicates that melatonin inhibited the apoptosis of cumulus cells by regulating the transcripts of proapoptosis, *Bax*, and antiapoptosis, *Bcl-2*. However, these changes were prevented in the presence of either of the selective MT2 antagonists (luzindole or 4-P-PDOT). These results suggest that melatonin enhanced cumulus expansion by regulating the expression of cumulus expansion and apoptosis genes via the MT2 receptor. In summary, the effects of melatonin on cumulus expansion, and the expression of cumulus expansion and apoptosis genes, appeared to be mediated predominantly by receptor binding through activation of the MT2 receptor, since luzindole or 4-P-PDOT, selective MT2 antagonists, blocked these effects.

Regarding the effects of melatonin with or without selective MT2 antagonists (luzindole or 4-P-PDOT) on nuclear maturation of porcine oocytes, no significant difference was observed in immature, degenerate, and MII oocyte rates between any treatment group and the control. The absence of any effects on nuclear maturation by melatonin is consistent with a previous study [24]. Also, neither of the MT2 antagonists, luzindole or 4-P-PDOT, affected nuclear maturation of porcine oocytes. Based on these results, we conclude that melatonin or MT2 antagonists have no effect on porcine oocyte nuclear maturation.

To investigate the subsequent embryo development of these treated in vitro matured oocytes, we performed a PA experiment. The results showed that melatonin significantly increased blastocyst formation rate and the total cell numbers of PA blastocysts. Blastocyst formation is an excellent indicator of culture system efficiency [38] and embryo development [39]. The total cell numbers of blastocysts is a routine criterion for evaluating embryo quality [40]. However, when either of the selective MT2 antagonists (luzindole or 4-P-PDOT) was added to the IVM culture medium simultaneously with melatonin, the stimulatory effects of melatonin were abolished. Therefore, these results suggest that the MT2 receptor mediates the stimulatory effects of melatonin on the development and quality of PA embryos.

In conclusion, this study provides the first evidence that the stimulatory effects of melatonin on cumulus expansion of porcine COCs, and subsequent embryo development of oocytes, are mediated by the MT2 receptor. These findings will be useful for potential clinical applications, suggesting that the MT2 receptor could be a promising therapeutic target for improving female reproductive capability. However, the specific mechanisms by which melatonin promotes porcine IVM through the MT2 receptor require more intensive research.

## 4. Materials and Methods

### 4.1. Chemicals

All chemicals and reagents used in this study were purchased from Sigma-Aldrich Chemical Company (St. Louis, MO, USA), unless otherwise stated.

### 4.2. Oocyte Recovery and In Vitro Maturation

Ovaries were obtained from prepubertal gilts at a local slaughterhouse, and were transported to the laboratory within 3 h after collection in physiological saline at 32–35 °C. Cumulus oocyte complexes (COCs) were aspirated from antral follicles (3 to 6 mm in diameter) using an 18-gauge needle fixed to a 10 mL disposable syringe, and allowed to settle in 50 mL conical tubes at 37 °C for 5 min. The supernatant was discarded and the sediment was washed three times in washing medium comprising tissue culture medium (TCM)-199 (Invitrogen, Carlsbad, CA, USA) containing 5 mM sodium hydroxide, 2 mM sodium bicarbonate, 10 mM HEPES, 0.3% polyvinyl alcohol, and 1% Pen-Strep (Invitrogen). In each experimental group, approximately 50 COCs were placed into an IVM medium comprising TCM-199 supplemented with 10 ng/mL epidermal growth factor, 0.57 mM cysteine, 0.91 mM sodium pyruvate, 10 μL/mL insulin transferrin selenium solution (ITS-A) 100× (Invitrogen), 10% porcine follicular fluid, 10 IU/mL eCG, and 10 IU/mL hCG. The COCs were cultured at 39 °C in a humidified atmosphere of 5% CO_2_. After 21–22 h of maturation culture with hormone, the COCs were washed three times in fresh hormone-free IVM medium, and then cultured in hormone-free IVM medium for another 21–22 h.

### 4.3. Detection of Melatonin Receptor 2 (MT2) in COCs by Immunofluorescence

Immature or in vitro matured COCs were washed three times in PBS containing 0.2% PVA, and fixed with 4% paraformaldehyde (*w*/*v*) in PBS for 1 h at room temperature. After washing three times in PBS with 0.2% PVA, COCs were permeated with 1% (*v*/*v*) Triton X-100 in PBS for 30 min. Then, COCs were washed three times in PBS with 0.2% PVA, and blocked with 2% bovine serum albumin (BSA) in PBS overnight at 4 °C. The COCs were incubated with a primary antibody for MT2 (1:200; ARP64072_P050; Aviva Systems Biology, San Diego, CA, USA) at 37 °C for 3 h. Subsequently, they were washed three times in PBS with 2% BSA, and then incubated with a secondary fluorescein isothiocyanate-conjugated anti-rabbit polyclonal antibody (1:200; ab6717, Abcam, Cambridge, UK) at 37 °C for 1 h (in darkness). After washing three times in PBS with 2% BSA, DNA was counterstained with 5 μg/mL Hoechst-33342 for 10 min, and samples were mounted on glass slides. Images were captured with an Eclipse TE200 confocal microscope (Nikon Corp., Tokyo, Japan).

### 4.4. Cumulus Expansion Assessment

The degree of cumulus expansion was evaluated by microscopic examination, according to Vanderhyden et al. [30]. A degree 0 indicates no detectable expansion, leaving a partially or fully denuded oocyte. A degree 1 indicates the minimum observable expansion, characterized by spherical and compacted cumulus cells around the oocyte. A degree 2 indicates only the outermost layers of cumulus cells expanded. A degree 3 indicates cumulus cells layers expanded up to, but not including the corona radiata. A degree 4 indicates the degree of complete expansion including the corona radiata.

### 4.5. Assessment of Nuclear Maturation

The COCs at 42 to 44 h after IVM were denuded by gently pipetting with 0.1% hyaluronidase in Tyrode’s albumin lactate pyruvate (TALP) medium. Then, the denuded oocytes were washed three times in TALP medium. The denuded oocytes were assessed under a microscope (TE2000-S, Nikon Corp.) and classified as follows: “immature”, oocytes without first polar body extrusion; “degenerate”, oocytes with ruptured membrane; “metaphase-II (MII)”, oocytes with polar body extrusion.

### 4.6. Parthenogenetic Activation (PA) of Oocytes

After 42 to 44 h of IVM culture, the COCs were denuded by gently pipetting with 0.1% hyaluronidase and washed three times in TALP medium. The MII stage oocytes were gradually equilibrated in activation medium consisting of 0.28 M mannitol, 0.5 mM HEPES, 0.1 mM CaCl_2_ and 0.1 mM MgSO_4_. Oocytes were transferred into a chamber connected to a BTX Electro-Cell Manipulator 2001 (BTX Inc., San Diego, CA, USA) and activated by a single direct current (DC) pulse of 1.5 kV/cm for 60 μs. Following electrical activation, the PA embryos were washed three times in fresh Porcine Zygote Medium-5 (PZM-5) (Funakoshi Corporation, Tokyo, Japan), transferred into wells containing 500 µL PZM-5 and cultured at 39 °C in a humidified atmosphere of 5% O_2_, 5% CO_2_ and 90% N_2_ for 7 days.

### 4.7. Embryo Evaluation and Total Cell Counts

The day when PA was performed was designated as Day 0. The embryos were evaluated under a stereomicroscope for cleavage on Day 2 (48 h), and for blastocyst formation on Day 7 (168 h). To determine the total cell numbers of blastocysts, Day 7 blastocysts were washed in TALP medium, and stained with 5 μg/mL Hoechst-33342 for 10 min. Following a final wash in TALP medium, stained blastocysts were mounted on glass slides in a drop of 100% glycerol, gently flattened with a cover glass, and observed under a fluorescence microscope (Nikon Corp.) at 400× magnification.

### 4.8. Gene Expression Analysis by Real-Time PCR

Total RNA was extracted from isolated cumulus cells using the easy-spin Total RNA Extraction Kit (iNtRON, Seoul, Korea). Total RNA were equalized for concentration across all the samples, and they were immediately stored at 80 °C until used real-time PCR. Complementary DNA (cDNA) was synthesized from total RNA using the RNA to cDNA EcoDry Premix, cDNA synthesis kit (Clontech Laboratories Inc., Mountain View, CA, USA). The cDNA was amplified in a 20 mL PCR reaction in a MicroAmp optical 96-well reaction plate (Applied Biosystems, Singapore) containing 1 μL cDNA, 0.4 μL (10 pmol/μL) forward primer, 0.4 μL (10 pmol/μL) reverse primer, 10 μL SYBR Premix Ex Taq (Takara, Otsu, Japan), and 8.2 μL of Nuclease-free water (Ambion, Austin, TX, USA). Each sample was performed in at least triplicate. The reactions were performed for 40 cycles, with the following parameters: denaturation at 95 °C for 15 s; annealing at 60 °C for 1 min; and extension at 72 °C for 1 min. Primer sequences, expected product sizes, and GenBank accession numbers for the real-time PCR analysis are presented in Table 1. The expression of each target gene was quantified relative to that of the endogenous control gene (*GAPDH*). The relative expression (R) was calculated using the following equation:

R = 2^−[Δ*C*t sample – Δ*C*t control]^

### 4.9. Experimental Design

We investigated whether the effects of melatonin on porcine IVM and subsequent embryo development were mediated by the MT2 receptor. Agents were added to the IVM medium during the entire maturation period of 44 h. The concentrations of melatonin (10^−9^ M) [24] and selective MT2 antagonists (luzindole and 4-P-PDOT) (10^−9^ M) [41] were set according to previous studies, and we designated six groups: (1) control; (2) melatonin; (3) luzindole; (4) 4-P-PDOT; (5) melatonin plus luzindole, or (6) melatonin plus 4-P-PDOT. In experiment 1, we investigated the presence of MT2 receptor protein in porcine COCs. In experiment 2, we investigated the degree of cumulus expansion and expression of genes associated with cumulus expansion, apoptosis and *MT2*. In experiment 3, we evaluated the oocyte maturation rate. In experiment 4, we assessed subsequent development of PA embryos.

### 4.10. Statistical Analysis

Statistical analyses were conducted with SPSS 22.0 (SPSS, Inc., Chicago, IL, USA). To determine differences among experimental groups, after normality and homoscedasticity test, the results were analyzed by a Kruskal–Wallis test for data with non-normal distribution or one-way ANOVA for data with normal distribution, followed by a Duncan’s multiple range test (when assumption of homoscedasticity was satisfied) or Dunnett’s T3 test (when assumption of homoscedasticity was not satisfied). The data are presented as means ± SEM. *p* values < 0.05 were considered to be significantly different.

## Figures and Tables

**Table 1:** List of real-time PCR primers.

Gene	Primer Sequences (5′-3′)	Product Size (bp)	GenBank Accession Number
*GAPDH*	F: GTCGGTTGTGGATCTGACCT R: TTGACGAAGTGGTCGTTGAG	207	NM_001206359
*Ptgs1*	F: CAACACGGCACACGACTACA R: CTGCTTCTTCCCTTTGGTCC	121	XM_001926129
*Ptgs2*	F: ACAGGGCCATGGGGTGGACT R: CCACGGCAAAGCGGAGGTGT	194	NM_214321
*Has2*	F: AGTTTATGGGCAGCCAATGTAGTT R: GCACTTGGACCGAGCTGTGT	101	AB050389
*Ptx3*	F: GGCCAGGGATGAATTTTAC R: GCTATCCTCTCCAACAAGTGA	185	NM_001244783
*Tnfaip6*	F: AGAAGCGAAAGATGGGATGCT R: CATTTGGGAAGCCTGGAGATT	106	NM_001159607
*Bax*	F: TGCCTCAGGATGCATCTACC R: AAGTAGAAAAGCGCGACCAC	199	XM_003127290
*Bcl-2*	F: AGGGCATTCAGTGACCTGAC R: CGATCCGACTCACCAATACC	193	NM_214285
*MT2*	F: CGGTCGTGTGCTTCTGTTACC R: AGCAGACGGCGAAGATCA	151	XM_013979266

F, forward; R, reverse.

## Abbreviations

Ptgs1	Prostaglandin-endoperoxide synthase 1
Ptgs2	Prostaglandin-endoperoxide synthase 2
Has2	Hyaluronan synthase 2
Ptx3	Pentraxin 3
Tnfaip6	Tumor necrosis factor α induced protein 6
Bcl-2	B cell leukemia/lymphoma 2
Bax	Bcl-2 associated X
